# First Trial of Male Circumcision against HIV

**DOI:** 10.1371/journal.pmed.0020391

**Published:** 2005-10-25

Over 3 million people died of AIDS last year and about 5 million others became infected with HIV, bringing the total number of people living with the infection to nearly 40 million. The continuing rise in the number of new cases makes it a priority to investigate all possible measures that might reduce the risk of infection, particularly—but by no means only—in Africa, which has 10% of the world's population but two-thirds of the world's people with HIV.

In many African tribal groups, men are circumcised, usually in late childhood or as teenagers, and this is an important part of their cultural identity. In other African ethnic groups, men are not circumcised. From observational studies dating back to the 1980s, it has become clear that HIV infection rates are greater in those groups where men were not circumcised. It has, however, remained a matter of speculation as to whether it is circumcision itself or some other difference in behavior that has a protective effect. What has been needed to settle this question is a randomized controlled trial (RCT) of the use of circumcision as a preventive intervention.

Auvert et al. have completed the first such trial in the Orange Farm area, a semiurban region close to Johannesburg, South Africa. They offered young, heterosexual uncircumcised men the chance to have the operation, explaining that half of those who came forward would be circumcised straightaway and the others (the control group) 21 months later. Some 3,000 men joined the study. It was planned that all the men would visit the research clinic four times during this 21-month period, and that they would be tested for HIV each time. They were instructed not to have sex for six weeks after the operation, and asked at each clinic visit to provide detailed information about their sexual activity. However, after 17 months, the number of new infections in the control group (49) was so much greater than the number in the treatment group (20) that it was considered unethical to continue the study, which was thus terminated early. Since infections were 60% lower in the treatment group, we now have strong evidence favoring the use of circumcision as part of prevention efforts.

Preliminary results from the Orange Farm study were presented in July at the 3rd International AIDS Society Conference in Rio de Janeiro, where there was considerable interest in the findings, although some cautionary notes were also sounded. Some observers have expressed concerns that the researchers did not tell participants of their HIV status, although they gave them every encouragement to attend a counseling and testing clinic in order to find this out. These and other issues are discussed in our accompanying Editorial (DOI: 10.1371/journal.pmed.0020293) and in two Perspectives, one by Peter Cleaton-Jones (DOI: 10.1371/journal.pmed.0020287), chair of the ethics committee that approved the study, and the second by Nandi Siegfried (DOI: 10.1371/journal.pmed.0020393), lead author of a Cochrane systematic review of male circumcision for prevention of heterosexual acquisition of HIV in men.

The authors have called for the promotion of male circumcision as part of AIDS prevention efforts in Orange Farm and in other parts of Africa. However, others will take a more cautious view, believing that the positive results of this one study must be confirmed by further studies. Such trials are currently under way in Kenya and Uganda. We must also remember that adult circumcision carries risks, especially if performed by medical personnel or traditional healers without proper training. A further concern is that circumcised men, considering themselves to be “protected,” might be more likely to engage in unsafe sex. Research is also needed to find out whether male circumcision has a preventive effect only on female-to-male transmission, or whether it may also reduce male-to-female transmission or male-to-male transmission. In addition, it will be important to determine the mechanism by which circumcision exerts its apparent protective effect.

Many questions do therefore remain, but in the words of one of the study's peer reviewers, this first RCT may come to be regarded as “a landmark paper” in HIV prevention.

